# Background-oriented schlieren imaging and tomography for rapid measurement of FUS pressure fields: initial results

**DOI:** 10.1186/2050-5736-3-S1-P68

**Published:** 2015-06-30

**Authors:** Michael Kremer, Charles Caskey, William Grissom

**Affiliations:** 1 Vanderbilt University, Nashville, Tennessee, United States

## Background/introduction

FUS pressure field mapping is important for dosimetry, quality assurance, and other uses. Hydrophone measurements are the current standard, and are accurate but costly and slow. As a simple low-cost alternative, background-oriented schlieren (BOS) imaging of ultrasound fields has been proposed.[1] In that technique, a predetermined image (usually a grid of lines or a random dot pattern) is placed on one side of a water tank and viewed from the other side, through the water and FUS pressure field. When the FUS is on, spatial variations in the water’s index of refraction are created that blur the image. Subtracting images with and without the FUS field provides a rapid visualization of it. The method has also been used to tomographically reconstruct air flow density.[2] The overall goal of the present work is to develop a low-cost BOS hardware system and BOS tomography acquisitions and reconstructions to enable rapid and cheap volumetric measurements of continuous-wave FUS fields. Here we present our current progress towards that goal.

## Methods

### Hardware Setup

**Figure 1:**
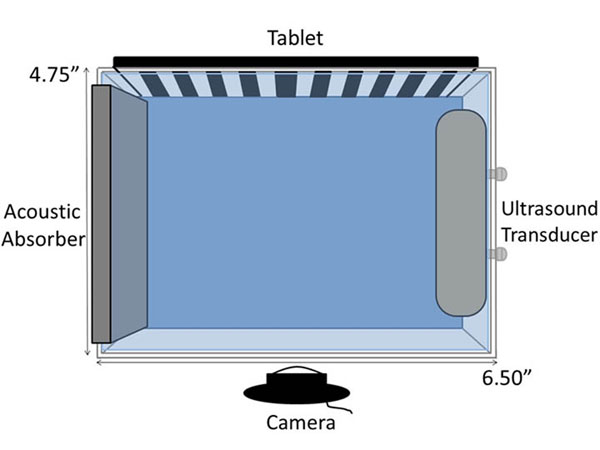
Hardware setup for BOS acquisitions.

Figure 1 illustrates our current experimental setup, comprising a water tank, a FUS transducer (Sonic Concepts HB 101, Bothell, WA), an Android tablet (Google Nexus 7) to display the background images, and a webcam (Logitech C920) to record the images. MATLAB (Mathworks) runs on a control PC to automate the acquisitions. In the next stage of the project the tablet and webcam will be mounted on a motorized gantry that rotates around the tank to acquire BOS images at multiple projection angles.

### Acquisitions

During acquisitions, the transducer was continuously pulsed at 1.1 MHz to generate a sound field with a peak negative pressure of 5.7 MPa at the focus. BOS acquisitions used background images comprising alternating white and black lines with varying positions, thicknesses and orientation angles.

### BOS Tomography Simulation

A 2D simulation was performed in MATLAB to validate the principles underlying BOS tomography, by implementing the forward model relating a spatially-varying index of refraction pattern to acquired BOS projection images, and a conjugate gradient reconstruction to invert that model. A parallel beam geometry was assumed.

## Results and conclusions

**Figure 2:**
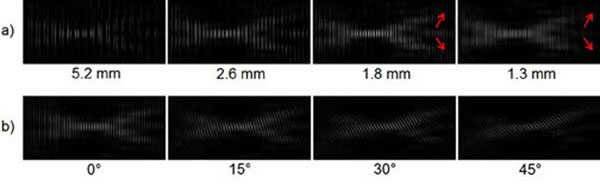
(a) Varying line widths, 0° angle. Thick widths can leave large gaps in the pattern, and thin widths can wash it out (arrows). (b) Varying angle of rotation, 1.8 mm line width. Different parts of the field pattern are emphasized at different angles.

**Figure 3:**
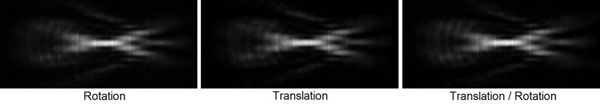
Comparison of BOS images summed across rotation angles, grid translations, and rotations+translations.

**Figure 4:**
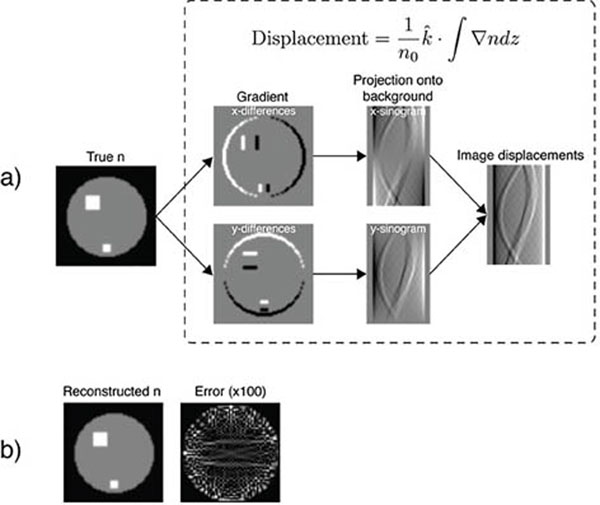
2D Simulation of BOS tomography. (a) The model relating the water index of refraction n to BOS projections. Elements of the displacement equation are embodied in the flow graph. (b) An accurate estimate of n can be reconstructed from the projections.

Figure 2 shows how BOS images depend on line widths and orientation. Figure 3 compares BOS images generated by summing across line positions and angles to eliminate stripe artifacts. Figure 4 illustrates the forward model and demonstrates that an accurate reconstruction can be achieved from data acquired under that model. Next we will translate our reconstruction to the true fan beam geometry and construct a motorized gantry to enable projections at multiple angles.
